# Extracellular Vesicles (EVs) Derived from Senescent Endothelial Cells Promote Platelet Activation

**DOI:** 10.3390/ijms27020869

**Published:** 2026-01-15

**Authors:** Whitney Venturini, Angel Cayo, Gabriel Diaz-Serrano, Sofia Sanhueza, Ricardo Huilcaman, Diego Méndez, Danitza Rebolledo-Mira, Catalina Silva-Pereira, Francisca Torres-Orellana, Felipe Troncoso, Carlos Escudero, Eduardo Fuentes, Andrew F. G. Quest, Claudio Valenzuela, Juan C. Tapia Amaro, Nelson E. Brown, Rodrigo Moore-Carrasco

**Affiliations:** 1 Departamento de Medicina Traslacional, Facultad de Medicina, Universidad Católica del Maule, Talca 3460000, Chile; wventurini@ucm.cl (W.V.); acayo@ucm.cl (A.C.); 2 Faculty of Health Sciences, University of Talca, Talca 3460000, Chile; gdiaz0890@gmail.com (G.D.-S.); danitza.rebolledo.ibi@gmail.com (D.R.-M.); catalinasilvapereira@gmail.com (C.S.-P.); franciscatorresorellana@gmail.com (F.T.-O.); 3 Cell Communication Laboratory, Center for Studies on Exercise, Metabolism and Cancer (CEMC), Institute of Biomedical Sciences (ICBM), Faculty of Medicine, University of Chile, Santiago 8380453, Chile; sofiasanhueza@ug.uchile.cl (S.S.); aquest@med.uchile.cl (A.F.G.Q.); 4 Advanced Center for Chronic Diseases (ACCDIS), Faculty of Medicine, University of Chile, Santiago 8380453, Chile; 5 Escuela de Tecnología Médica, Facultad de Ciencias de la Salud, Universidad Bernardo O’Higgins, Santiago 8320000, Chile; ricardo.huilcaman@ubo.cl; 6 MIBI: Interdisciplinary Group on Mitochondrial Targeting and Bioenergetics, Department of Clinical Biochemistry and Immunohematology, Thrombosis Research Center, Medical Technology School, Faculty of Health Sciences, Universidad de Talca, Talca 3460000, Chile; diego.mendez@utalca.cl (D.M.); edfuentes@utalca.cl (E.F.); 7 Center for Medical Research, Medical School, University of Talca, Talca 3460000, Chile; cvalenzuela@utalca.cl; 8 Vascular Physiology Laboratory, Group of Research and Innovation in Vascular Health (GRIVAS Health), Basic Sciences Department, Faculty of Sciences, Universidad del Bio-Bio, Chillan 3780000, Chile; fetronc@gmail.com (F.T.); cescudero@ubiobio.cl (C.E.); 9 Stem Cells and Neuroscience Research Center, University of Talca Medical School, Talca 3460000, Chile; juantapia@utalca.cl

**Keywords:** thrombosis, endothelial senescence, Doxorubicin, extracellular vesicles, platelet activation, cancer therapy, hemostasis

## Abstract

Thrombotic cardiovascular diseases are frequent side effects of cancer therapy with cytotoxic drugs such as Doxorubicin. Endothelial cell senescence is emerging as a critical mechanism underlying endothelial dysfunction in this context. Senescent cells, although unable to proliferate, secrete bioactive molecules that alter the tissue microenvironment, a feature known as the senescence-associated secretory phenotype (SASP). Besides soluble molecules, senescent cells also release extracellular vesicles (EVs). Previous studies indicate that senescent endothelial cells produce a secretome that promotes platelet activation; however, the contribution of EVs remains unclear. Here, we show that human microvascular endothelial cells (HMEC-1) exposed to Doxorubicin undergo senescence, display endothelial dysfunction, and release EVs. We found no differences in the concentration or size distribution of EVs from senescent and non-senescent cells. Nevertheless, EVs from senescent HMEC-1 promoted platelet activation more strongly than EVs from control cells. Notably, EVs alone did not induce platelet aggregation, suggesting that soluble factors are also required to support platelet-dependent hemostasis. These findings reveal that EVs from senescent endothelial cells contribute to platelet activation, a process that may favor thrombosis in patients receiving Doxorubicin-based chemotherapy.

## 1. Introduction

Every year, about one in six deaths worldwide is due to cancer [1]. While the overall cancer mortality rate has decreased in recent years—due, in part, to early detection and improved treatments—the incidence of cancer continues to increase, in part as a consequence of aging [2]. Chemotherapeutic drugs are often used to treat disseminated malignancies or as adjuvants preceding or following surgery or radiotherapy. Importantly, patients with good responses to chemotherapy, who may even survive cancer, often develop life-threatening cardiovascular diseases related to endothelial dysfunction. Indeed, cardiovascular diseases, including thrombosis, are currently the leading cause of death among cancer survivors [3]. The risk of thrombosis varies depending on cancer type, stage, and the treatments received [4,5]. Patients with pancreatic, brain, and lung cancer have a significantly higher risk of developing thrombosis compared to those with breast and prostate cancer [6]. The risk of thrombosis can increase by up to 20% due to the aggressiveness of the cancer and the development of metastasis [5]. Furthermore, specific chemotherapy treatments can further raise the risk of thrombosis, contributing to the overall higher incidence observed in these patients [3]. Nonetheless, despite the well-known association between cancer therapies and the development of cardiovascular diseases, the mechanisms underlying prothrombotic states remain unknown.

Among chemotherapeutic drugs, Doxorubicin is widely used, in combination with other drugs, in the treatment of various types of cancer, including carcinomas, sarcomas, and hematological malignancies [7]. Despite its versatility, injury to normal tissues often complicates Doxorubicin-based therapies, thus diminishing the patients’ quality of life during or after treatment [7]. While the heart constitutes a relatively specific off-target of Doxorubicin, it can also affect other organs, such as the brain, the kidney, and the liver [8]. In addition, Doxorubicin has been reported to induce premature aging in murine models [9] and endothelial dysfunction [10]. Whether the effects of Doxorubicin on endothelial cells are due to the induction of cellular senescence [11,12] or other forms of endothelial damage that may be accompanied by production of the endothelium-dependent vasodilator nitric oxide [13] and plasminogen activator inhibitor-1 (PAI-1) [14] remains incompletely understood. It is now widely accepted that the mechanisms through which Doxorubicin induces cell damage and/or senescence are multiple and primarily include DNA intercalation, inhibition of topoisomerase II, and free radical generation [15]

Cellular senescence is a stable and permanent form of cell cycle arrest, characterized by the acquisition of distinct morphological and biochemical properties [16,17]. Cellular senescence can be triggered by various intrinsic and extrinsic stimuli, including telomere shortening (a form of cellular senescence known as replicative senescence), oncogene activation (a form of cellular senescence known as oncogene-induced senescence, OIS) and drugs (also known as drug-induced senescence or therapy-induced senescence) [16]. Although first described as an in vitro phenomenon, cellular senescence also occurs in vivo, with beneficial or detrimental consequences for tissue homeostasis depending on the cellular and tissue context. For example, cellular senescence represents an intrinsic barrier to tumorigenesis [18]. However, the accumulation of senescent cells in tissues may also promote tumor initiation or progression in a non-cell autonomous manner [16].

Notably, the accumulation of senescent cells has also been linked to other chronic diseases, including those affecting the cardiovascular system [19]. In the pathological setting, the mechanisms explaining the detrimental effects of senescence seem to be related to the ability of senescent cells to synthesize and secrete a variety of bioactive molecules, many of which can in turn impinge on tissue microenvironments, a feature referred to as the senescence-associated secretory phenotype (SASP) [17].

In addition to soluble molecules, senescent cells can produce and release extracellular vesicles (EVs) [20,21]. EVs are small, membranous structures that play critical roles in intercellular communication through their ability to transport and deliver a variety of cargoes [21]. Based on their size and biogenesis, at least two main subtypes of EVs have been characterized: exosomes, which are typically between 30 and 150 nm in diameter, and microvesicles, which are larger in size, ranging from 100 to 1000 nm in diameter [22]. While exosomes originate intracellularly in multivesicular bodies (MVBs), microvesicles are the result of budding and compression directly outward from the plasma membrane [22].

It has been shown that senescent cells can produce and release higher amounts of EVs when compared to their non-senescent counterparts [20]. EVs derived from senescent cells contain diverse cargoes—proteins, nucleic acids, and lipids—whereby the specific composition varies depending on the cell type and the senescence-inducing stimulus [21]. Since these cargoes can be transferred to neighboring cells in a paracrine manner, EVs derived from senescent cells likely alter the functional state of neighboring non-senescent cells [23]. Interestingly, EVs derived from senescent cells can promote a procoagulant state [24], favoring the formation of thrombi or negatively affecting the function of blood vessels [25].

Among cells in blood vessels that may become senescent in the context of cancer chemotherapy, endothelial cells are particularly attractive. Endothelial cells modulate vascular resistance by synthesizing and releasing vasoactive mediators, balancing vasodilation induced by nitric oxide and prostacyclin with vasoconstriction mediated by endothelins [26]. In addition, the endothelium actively participates in hemostasis, maintaining an exquisite balance between prothrombotic and antithrombotic capabilities [27]. This function is complemented by its role in inflammation and immune response through the regulation of adhesion molecules and the secretion of cytokines [28]. Interestingly, as senescent endothelial cells display pro-inflammatory and pro-coagulant features, including the expression of adhesion factors and procoagulant molecules [12,29], the emergence of these cells in vivo may be a key event explaining the prothrombotic effects of chemotherapeutic drugs such as Doxorubicin. Thus, a better understanding of the effects of soluble secreted factors and EVs released by Doxorubicin-driven senescent endothelial cells on thrombus formation is needed.

We have previously reported that senescent human microvascular endothelial cells (HMEC-1) produce a secretome that triggers platelet activation [12]. Altogether, this study aimed to determine the role of extracellular vesicles released by Doxorubicin-induced senescent endothelial cells in the activation of platelets. We demonstrate that these vesicles enhance platelet activation, supporting a novel mechanism through which chemotherapy may favor thrombosis in cancer patients.

## 2. Results

### 2.1. Senescent Endothelial HMEC-1 Cells Display Reduced Expression of Markers of Endothelial Cell Function

**Figure 1:**
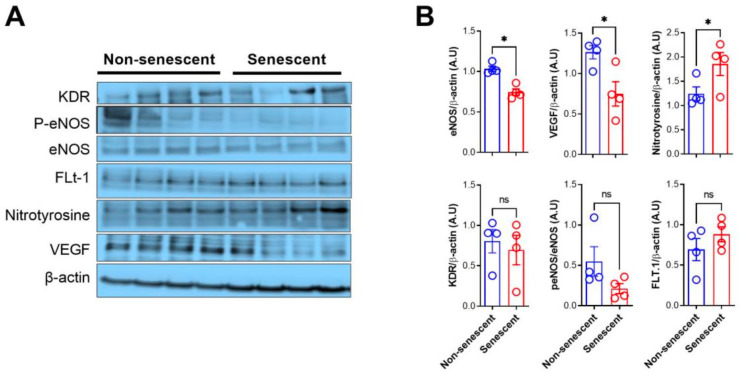
Changes in the expression of markers of endothelial function in senescent HMEC-1 cells. (**A**) Representative images and densitometric analysis (**B**) of immunoblots for the detection of markers of endothelial function in senescent and non-senescent endothelial HMEC-1 cells. β-actin was used as a loading control. In panel (**B**), the upper charts depict relative protein levels of eNOS (endothelial nitric oxide synthase), VEGF (Vascular Endothelial Growth Factor), and nitrotyrosine, while the lower charts show KDR (vascular endothelial growth factor receptor 2), the p-eNOS/eNOS ratio, and FLT-1 (vascular endothelial growth factor receptor 1). Values are expressed as the mean ± SEM. Each circle on the bars indicates an individual replicate (n = 4 independent samples). Statistical comparisons were made using the one-tailed Mann–Whitney test with GraphPad 9. ns = not significant, * = *p* < 0.05. A.U. = arbitrary densitometric units.

Induction of cellular senescence in HMEC-1 cells was achieved by exposing them to 0.05 μM Doxorubicin for 72 h (Figure S1A) [12]. Our model of Doxorubicin-induced senescence was confirmed by detecting β-galactosidase (β-Gal) enzyme activity at suboptimal pH (pH 6.0), a widely used marker of cellular senescence (Figure S1B). Next, we assessed the functional status of senescent endothelial HMEC-1 cells. Previous studies have shown that senescent cells of endothelial origin show various changes indicative of endothelial dysfunction [30]. We therefore determined by Western blotting the levels of proteins and their derivatives, which are essential for endothelial function. These included KDR (also known as vascular endothelial growth factor receptor 2, VEGF-R2), eNOS (endothelial nitric oxide synthase), peNOS (phosphorylated version of eNOS), FLT-1 (also known as endothelial growth factor receptor 1, VEGF-R1), nitrotyrosine, and VEGF (Vascular Endothelial Growth Factor). As shown in Figure 1, there was a significant decrease in eNOS and VEGF levels, as well as a significant increase in nitrotyrosine levels, in senescent HMEC-1 cells (Doxorubicin-treated) when compared to the non-senescent HMEC-1 (1×PBS-treated) controls (Figure 1A,B). Importantly, nitrotyrosine levels have been linked to oxidative stress, a known risk factor for cardiovascular diseases [31]. On the other hand, although there were minor differences between both groups for the proteins KDR, peNOS, and FLT-1, these differences were not statistically significant (Figure 1A,B). Thus, although further work will be necessary to determine if these changes are accompanied by alterations in the barrier function of endothelial cells, our data reveal differences in the functional status of senescent endothelial HMEC-1 cells compared to their non-senescent counterparts, in agreement with results from our previous studies [32,33].

### 2.2. Doxorubicin-Induced Senescent Endothelial HMEC-1 Cells Liberate EVs

Accumulating evidence indicates that senescent cells, in addition to synthesizing and secreting soluble factors, also generate EVs that, depending on their composition (cargo), may be able to alter the tissue microenvironment and neighboring cells [20,21]. To isolate and characterize the EVs present in the secretome derived from senescent and non-senescent endothelial HMEC-1 cells, we collected 100 mL of conditioned medium (CM) for each experimental condition. Conditioned media were further concentrated, and the EVs were then purified using size exclusion columns. Isolated EVs were then characterized by nanoparticle tracking analysis (NTA), scanning electron microscopy (scanning electron microscope, SEM), and Western blotting (WB), essentially following the guidelines established by the International Society for Extracellular Vesicles [34].

**Figure 2:**
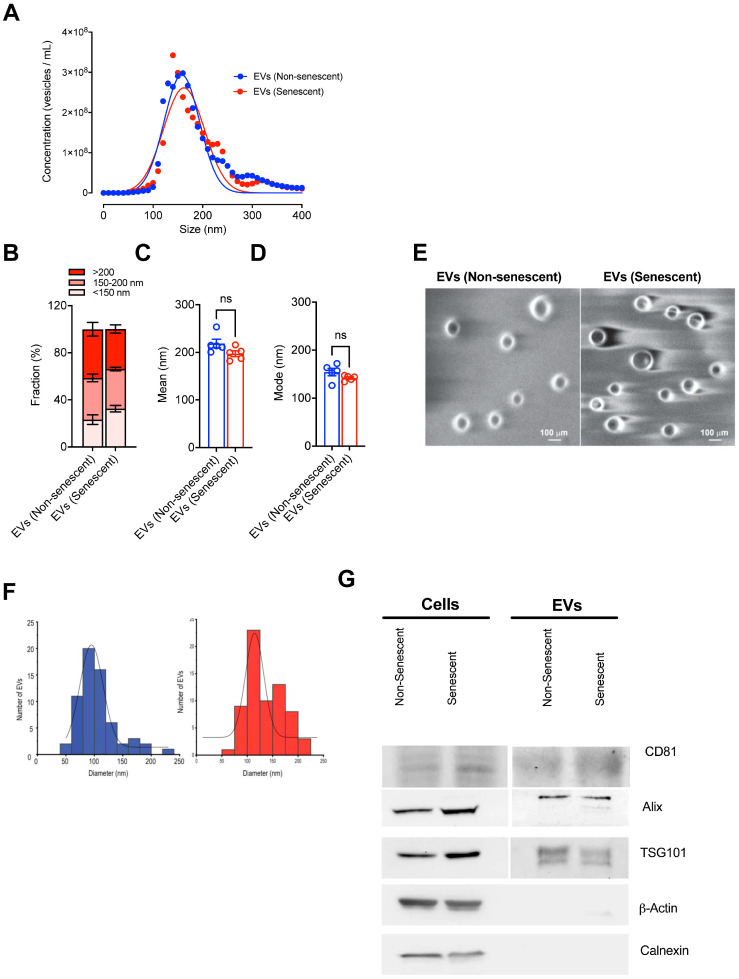
Senescent and non-senescent endothelial HMEC-1 cells release extracellular vesicles (EVs). (**A**) Concentration of EVs released by senescent and non-senescent HMEC-1 cells according to size. (**B**) Percentage of EVs released by senescent and non-senescent HMEC-1 cells that fell into the size ranges indicated (>200 nm, 150–200 nm, and <150 nm fractions). (**C**) Mean size of EVs released by senescent (red) and non-senescent (blue) HMEC-1 cells determined by nanoparticle tracking analysis (NTA). (**D**) The most frequently occurring size value (mode) of EVs derived from senescent (red) and non-senescent (blue) HMEC-1 cells according to NTA. (**E**) Representative images obtained by scanning electron microscopy (SEM) analyses of EVs released by senescent and non-senescent HMEC-1 cells. A 100 μm magnification bar is shown for each image. (**F**) Histogram profiles of the number of EVs versus their diameter (nm) are shown. (**G**) Western blotting for the detection of CD81, ALIX and TSG101 proteins (exosome markers used as positive controls for exosomal EVs), β-actin, and calnexin (negative controls). A representative image of three independent experiments is shown. EVs: extracellular vesicles; Cells: cell lysates. Values are expressed as mean ± SEM. Circles represent individual observations. For (**B**), comparisons were made by two-way ANOVA using the Bonferroni post-test. For (**C**,**D**), comparisons were made using the two-tailed Welch’s *t*-test. All analyses were computed by GraphPad 9.

Nanoparticle tracking analyses (NTA) allowed us to determine the concentrations per mL, the size, and the mean and mode of the size values for the EVs present in the conditioned media derived from non-senescent and senescent HMEC-1 cells. First, we analyzed the concentrations of EVs produced by senescent and non-senescent HMEC-1 cells. For EVs from non-senescent (Figure 2, blue lines or dots) and senescent (Figure 2, red lines or dots) cells, concentrations of 3.34 × 10^7^ ± 1.56 × 10^7^ and 3.19 × 10^7^ ± 1.37 × 10^6^ vesicles per mL, respectively, were detected with no statistically significant differences between the two groups (Figure 2A). Next, the size distribution of EVs for each group was analyzed. For EVs present in conditioned media derived from non-senescent HMEC-1 cells, 23.09 ± 4.06% of the vesicles were smaller than 150 nm, while 35.51 ± 3.33% and 41.38 ± 5.78% were 150–200 nm or larger than 200 nm, respectively (Figure 2B). The analysis of EVs present in conditioned media derived from senescent HMEC-1 cells revealed that 32.45 ± 2.80% of the vesicles were smaller than 150 nm. In comparison, 33.74 ± 1.46% and 33.97 ± 3.60% of the vesicles were 150–200 nm or larger than 200 nm, respectively (Figure 2B). Overall, although there were minor differences in the sizes between groups, they did not reach significance. We were also unable to detect significant differences between the mean diameter values of EVs derived from senescent and EVs derived from non-senescent HMEC-1 cells (mean of 218.6 ± 9.08 nm for EVs derived from non-senescent cells and 197.3 ± 5.98 nm for EVs derived from senescent HMEC-1 cells) (Figure 2C). Similarly, the modes, the most frequently occurring size value, of vesicles derived from non-senescent (Figure 2D, blue bar) and senescent HMEC-1 (Figure 2D, red bar) cells were 154.34 ± 7.73 nm and 141.8 ± 2.39 nm, respectively (Figure 2D). Overall, while both the mean and the mode of size values were lower for EVs released by senescent HMEC-1 cells, these differences did not reach statistical significance.

In order to complement our analyses, EVs released by senescent and non-senescent endothelial HMEC-1 cells were visualized by scanning electron microscopy (SEM, see methods). Confirming the results obtained by NTA, the EVs observed were spherical with an approximate diameter of 100 nm for both experimental conditions, a size within the expected range of EVs (Figure 2E,F).

Finally, the characterization of EVs released by senescent and non-senescent HMEC-1 cells was completed by Western blotting analysis of specific marker proteins (www.exocarta.org) present either in whole cell lysates or EVs purified from conditioned medium derived from senescent or non-senescent HMEC-1 cells (Figure 2G). EVs derived from both senescent and non-senescent HMEC-1 cells were enriched for proteins involved in the biogenesis of EVs (ALIX and TSG101). As expected, EVs lacked the endoplasmic reticulum protein Calnexin, as well as β-Actin (Figure 2G). These data suggest that both senescent and non-senescent HMEC-1 cells liberate EVs suitable for functional assays.

### 2.3. EVs Derived from Endothelial HMEC-1 Cells Do Not Alter the Viability of Human Platelets

**Figure 3:**
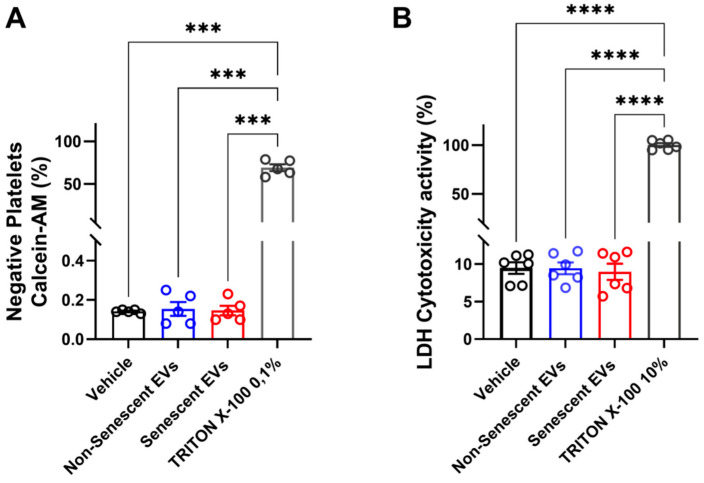
The cytotoxic effect of EVs derived from endothelial HMEC-1 cells on human platelets. (**A**) Washed platelets were incubated with EVs derived from non-senescent and senescent HMEC-1 cells, and platelet viability was assessed by flow cytometry using Calcein-AM. Platelets negative for CD61 and Calcein-AM staining were considered non-viable. (**B**) Washed platelets were incubated with EVs derived from non-senescent and senescent HMEC-1 cells, and LDH release was tested using the LDH cytotoxicity assay kit (Cayman Chemical, Ann Arbor, MI, USA). Data are presented as mean ± SEM. For panel (**A**), values correspond to the percentage of Calcein-AM-negative (non-viable) platelets. For panel (**B**), values correspond to the percentage of LDH released relative to total LDH activity measured in Triton X-100-lysed platelets (defined as 100% cytotoxicity). Each circle shows individual observations. Non-senescent EVs, blue bars; senescent EVs, red bars. Vehicle, black bars. Positive controls, gray bars. Comparisons were made with Welch ANOVA using Dunnett’s T3 as a post-test. All analyses were computed by GraphPad 9. For statistical differences: *** = *p* < 0.005; **** = *p* < 0.0001.

To assess the impact of EVs derived from endothelial HMEC-1 cells on platelet viability, two classical cytotoxicity assays were performed, namely, Calcein-AM incorporation (a marker that emits fluorescence once incorporated into viable cells) and LDH (lactate dehydrogenase) release [35,36]. To this end, washed platelets were incubated with EVs purified from media conditioned by non-senescent and senescent endothelial cells. For these assays, we used amounts of EVs corresponding to 13 ng/µL. Platelets negative for CD61 and Calcein-AM were considered non-viable for the Calcein-AM incorporation assays. As shown in Figure 3, EVs harvested from non-senescent endothelial cells (0.154 ± 0.03%) and EVs generated from senescent HMEC-1 cells (0.146 ± 0.02%) did not show a significant increase in the Calcein-AM negative platelet population when compared with platelets that were not exposed to any conditioned medium (Figure 3A). Values in parentheses indicate mean ± SEM of the percentage of Calcein-AM-negative (non-viable) platelets. Platelets treated with 0.1% Triton X-100 were used as a positive control for cell death (69.02 ± 3.99%; Figure 3A). These assays suggested that EVs released by endothelial cells, both senescent and non-senescent, are not cytotoxic to platelets. To corroborate this result, the release of LDH from human platelets after being incubated in the presence of EVs was evaluated. As previously, no significant changes in LDH release were observed when washed platelets were incubated in the presence of EVs collected from non-senescent (9.43 ± 0.78%) or senescent (8.96 ± 1.07%) HMEC-1 cells. Here, LDH values are expressed as the percentage of total LDH activity measured in platelets lysed with Triton X-100 (defined as 100% cytotoxicity). Platelets previously incubated in 10% Triton X-100 were used as positive controls for cell death (100 ± 1.89%) (Figure 3B) [36]. These results showed that EVs produced and released by endothelial HMEC-1 cells did not have any cytotoxic effects on human platelets.

### 2.4. EVs Derived from Senescent Endothelial HMEC-1 Cells Promote Platelet Activation

**Figure 4:**
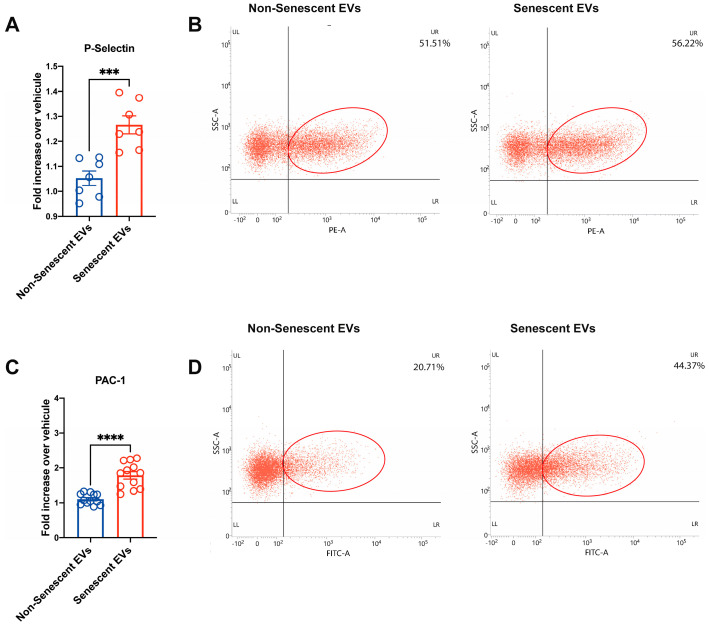
Flow cytometric analyses for the detection of P-selectin and activated GIIb/IIIa (PAC − 1) on the surface of platelets following their incubation with EVs derived from non-senescent and senescent HMEC-1 cells. (**A**) Platelet activation was detected as the expression of P-selectin on the surface of platelets following their incubation with the indicated EVs. (**B**) Representative dot plots for the detection of P-selectin. (**C**) Expression of activated GPIIb/IIIa (PAC − 1) on the surface of platelets following their incubation with the indicated EVs. (**D**) Representative dot plots for the detection of activated GPIIb/IIIa (PAC − 1). Non-senescent EVs (blue bars) and senescent EVs (red bars), at a concentration of 13 ng/uL of protein. The data were normalized to values in the control condition (i.e., treated with vehicle). Values are expressed as mean ± SEM. Circles show individual observations. Two-tailed Welch’s *t*-test comparison of the data. All analyses were computed by GraphPad 9. For statistical differences: *** = *p* < 0.005; **** = *p* < 0.0001.

Little is known about the direct effect of factors released by senescent endothelial cells on platelet function. Our previous work revealed that soluble factors produced by senescent endothelial cells induced platelet adhesion, activation, and aggregation [12]. In this study, we focused on the role of EVs released by senescent or control HMEC-1 cells in platelet activation. We found that the expression of the markers P-selectin and activated GPIIb/IIIa (also known as PAC-1) was significantly increased on the surface of platelets that had been exposed to EVs from senescent cells (Figure 4). To this end, we pre-incubated washed platelets with EVs obtained from senescent or non-senescent endothelial cells at a concentration of 13 ng/µL. As shown in Figure 4A, EVs derived from senescent HMEC-1 cells significantly increased P-selectin expression (1.26 ± 0.02) in platelets, compared to EVs derived from non-senescent HMEC-1 cells (1.05 ± 0.03) (Figure 4A). Similarly, EVs isolated from senescent HMEC-1 cells increased PAC-1 expression on the platelet surface (1.78 ± 0.10) compared to values observed with EVs derived from non-senescent HMEC-1 cells (1.10 ± 0.04) (Figure 4B). Our results indicate that EVs from senescent endothelial cells are more potent in inducing platelet activation compared to those from non-senescent cells.

We examined whether EVs released by senescent HMEC-1 cells altered platelet aggregation. To investigate, we treated platelets with EVs generated by control and senescent cells. Our findings revealed no significant differences between the groups. While platelet activation is essential for initiating the formation of aggregates, stable aggregation does not always require complete activation. Initial activation may be sufficient for the expression of specific markers, such as P-selectin and PAC-1. Additionally, in certain circumstances, platelet aggregates can form independently of full platelet activation [37]. These results imply that although EVs harvested from senescent endothelial cells can upregulate markers of platelet activation, additional factors may be necessary to induce those activated platelets to aggregate. However, additional assays are necessary to further elucidate the role of EVs in this process.

Additionally, we assessed the impact of EVs derived from both senescent and non-senescent endothelial cells on the coagulation cascade. Previous studies (D Wiley et al., 2019) reported no differences in activated partial thromboplastin time (aPTT) and prothrombin time (PT) assays using conditioned media from senescent and non-senescent cells [38]. Consistent with Wiley’s findings, our results revealed no significant differences between the senescent and non-senescent EV-treated groups (Figure S2A,B). aPTT and PT values remained unchanged for both groups, indicating that EVs from senescent endothelial cells do not impact either the intrinsic or extrinsic pathways of the coagulation cascade.

Finally, our results suggest that EVs produced and secreted by senescent endothelial HMEC-1 cells may play a role in platelet activation. This, in turn, may contribute to the development of thrombosis and cardiovascular diseases in cancer patients following Doxorubicin-based chemotherapeutic treatments.

## 3. Discussion

Over recent decades, the overall cancer survival rates have shown remarkable improvements due to early detection and better therapeutic approaches [2]. However, these improvements have led to yet another problem, the rise of long-term effects of chemotherapeutic drugs [39]. The harmful side effects of chemotherapeutic drugs are now widely recognized [40]. It is also well known that cancer chemotherapy is associated with accelerated aging in younger individuals [2] or can trigger a frailty state in the elderly [41].

Among chemotherapeutic drugs, anthracyclines are notorious for their off-target effects in the heart and blood vessels [42]. Thus, cancer patients undergoing treatment with the anthracycline Doxorubicin can develop cardiac toxicity, which includes the development of arrhythmias and dilated cardiomyopathy [42]. Interestingly, 4–20% of these patients also develop thrombotic events [43]. For example, Doxorubicin-based chemotherapy has been linked to an increased risk of developing venous thrombosis in cancer patients [44]. These thrombotic effects are presumably because of Doxorubicin on vascular endothelial cells, platelets, and/or monocytes [45]. So far, however, little is known about the mechanisms through which Doxorubicin or other chemotherapeutic drugs induce thrombosis.

Using in vitro approaches, we and others have been interested in understanding the mechanisms through which factors released by senescent cells—driven by treatments with Doxorubicin or other cancer drugs—modulate platelet function [12,38,46]. Previously, we have shown that factors secreted by senescent tumor cells collaborate with platelets to enhance the pro-invasive features of non-senescent tumor cells [46]. Similarly, components of the secretome derived from Doxorubicin-driven senescent endothelial HMEC-1 cells induce platelet activation and aggregation [12]. However, it was unclear whether platelet activation in these environments was entirely a consequence of the action of soluble factors released by senescent cells or, alternatively, EVs derived from senescent cells could also contribute to such effects. In this context, it was recently shown that cells undergoing replicative senescence can produce and release EVs [20]. However, their effect on platelet function remained unexplored.

In this study, we complemented previous observations by showing that EVs derived from endothelial HMEC-1 cells undergoing senescence following Doxorubicin exposure can also induce platelet activation. This and previous work support the notion that senescence-related mechanisms may help explain the prothrombotic effects of Doxorubicin and other anti-cancer drugs [39]. Similarly, the accumulation of senescent endothelial cells that occurs in the context of physiological aging may also lead to increased activation of platelets [29].

Before testing the effects of EVs on platelets, we set out to purify and characterize the EVs derived from senescent and non-senescent HMEC-1 cells. Importantly, we did not detect any differences in the amounts of EVs released by senescent and non-senescent HMEC-1 cells (Figure 2A). Similarly, while there was some enrichment of EVs of less than 150 nm in diameter in conditioned media derived from senescent HMEC-1 cells (Figure 2B), the differences in size distribution did not reach statistical significance. Therefore, these results differ from previous studies that did show an increase in exosome production by endothelial cells undergoing replicative senescence [20]. Moreover, the amount of EVs purified from each group was reduced when compared to the amount of EVs isolated from other endothelial cell lines [20]. These findings can be explained by differences in the cell type studied. Moreover, EVs, regardless of senescence status, did not affect the viability of platelets at a concentration of 13 ng/µL (Figure 3A,B).

We also tested the effects of EVs on platelets using the platelet activation surface markers P-selectin and PAC-1 (GPIIbIIIa) (Figure 4). Our results indicate that EVs derived from senescent HMEC-1 cells trigger platelet activation by promoting the plasma membrane translocation of P-selectin from alpha granules (Figure 4). P-selectin participates in the adhesion of platelets to the subendothelial matrix upon loss of integrity or dysfunction of the endothelial monolayer [47]. Similarly, EVs derived from senescent HMEC-1 cells triggered a transition of the integrin GPIIbIIIa (alpha2 beta3 integrin) from its inactive to an active conformation (Figure 4). The activation of GPIIb/IIIa is necessary for platelet aggregation [48]. Interestingly, unlike experiments in which platelets were exposed to whole conditioned media derived from senescent endothelial HMEC-1 cells [12], platelets exposed to EVs purified from these cells did not undergo aggregation, suggesting that other, presumably “soluble” factors are necessary to complete the process. Thus, EVs seem to favor platelet transition towards an activated state, but other factors are required to complete the process of aggregation. Moreover, no differences were observed in the coagulation assay (Figure S1), indicating that EVs alone are not sufficient to induce coagulation. This observation is consistent with findings by others, who also reported no effects of senescent conditioned media on coagulation [38]. Therefore, EVs appear to drive platelets towards an activated state, but other factors are required to complete the platelet aggregation process. Further experiments are needed to fully understand the mechanisms behind platelet aggregation and coagulation in these settings.

**Figure 5:**
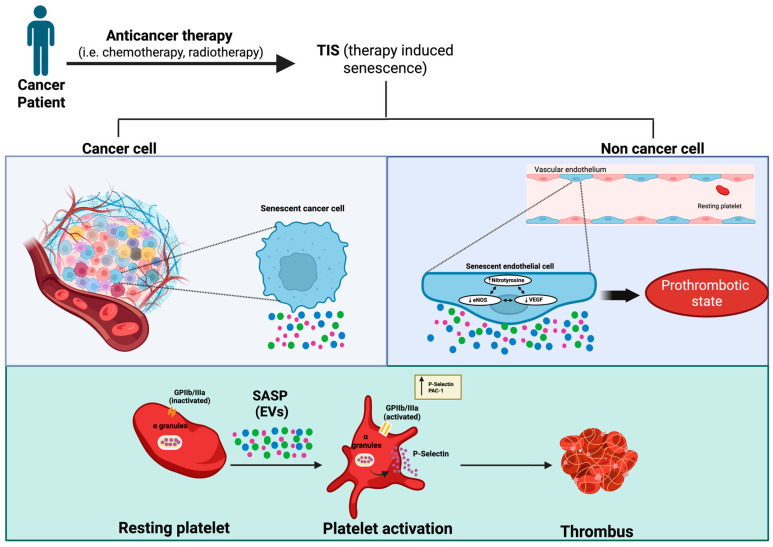
The proposed mechanism linking therapy-induced senescence (TIS) to platelet activation and cardiovascular diseases. “Created with BioRender.com”.

Altogether, although our study suffers from certain limitations, such as the use of a single line of endothelial cells and the lack of mechanistic details, we believe that, in general, this is one of the first works that relates extracellular vesicles released by senescent endothelial cells and the primary thrombosis process. These findings highlight extracellular vesicles as active mediators of platelet activation in the context of Doxorubicin-induced endothelial senescence, opening the field for potential mechanistic links between chemotherapy and thrombosis in cancer patients. Future studies should aim to characterize the molecular cargo of these vesicles and to validate their role in vivo, which could open avenues for therapeutic intervention or biomarker development (Figure 5).

## 4. Materials and Methods

### 4.1. Cell Culture

The human microvascular endothelial cell line (HMEC-1) was provided by Dr. Fernando Delgado from Universidad Católica del Maule (UCM), Talca, Chile, and described previously [12]. HMEC-1 cells were propagated in 10 cm diameter sterile culture dishes or multi-well plates, depending on the specific experiment to be performed. HMEC-1 cells were cultured in MCDB-131 medium (Gibco, Thermo Scientific, South Logan, UT, USA) supplemented with 10% Fetal Bovine Serum (FBS; Hyclone, Thermo Scientific, South Logan, UT, USA), 10 ng/mL Epidermal Growth Factor (EGF) (Gibco, Thermo Scientific, South Logan, UT, USA), 1 µg/mL Hydrocortisone (Gibco, Thermo Scientific, South Logan, UT, USA), 2 mM L-Glutamine (Gibco, Thermo Scientific, South Logan, UT, USA), 25 μg/mL Amphotericin B (InvivoGen, San Diego, CA, USA), 25 μg/mL Gentamicin (Hyclone, Thermo Scientific, South Logan, UT, USA) and 5 μg/mL Plasmocin (InvivoGen, San Diego, CA, USA). Cells were maintained at 37 °C and 5% CO_2_ in a humidified chamber.

### 4.2. Induction of Cellular Senescence

For the detection of cellular senescence, 0.7 × 10^5^ (1 × PBS-treated control group) and 1.2 × 10^6^ (Doxorubicin-treated group) HMEC-1 cells were first seeded on 100 mm dishes. The next day, cells were exposed to 0.05 µM of the drug Doxorubicin (MP Biomedicals, LLC, Santa Ana, CA, USA) or vehicle (1 × PBS, Phosphate-Buffered Saline, Hyclone, Thermo Scientific, South Logan, UT, USA) for 72 h, following a well-established and previously described protocol [12]. This protocol has been widely used and validated in the field. Subsequently, 72 h after treatment with Doxorubicin, induction of cellular senescence was confirmed through histochemical detection of beta-galactosidase enzyme activity at suboptimal pH (pH 6.0), essentially as previously described [46].

### 4.3. Harvesting of Conditioned Media

HMEC-1 cells (0.7 × 10^5^ cells for the 1 × PBS-treated control group; 1.2 × 10^6^ for the Doxorubicin-treated group) were seeded on 100 mm dishes. The next day, the cells began to be exposed to culture media supplemented with 0.05 µM Doxorubicin in 1 × PBS, or 1 × PBS alone. After 72 h of exposure, the cells were washed three times in 1 × PBS, and 4 mL of serum-free medium was added. Twenty-four hours later, conditioned media were collected and centrifuged at 2000× *g* for 30 min at 4 °C (D3024R microcentrifuge, SCILOGEX, EEUU, Rocky Hill, CT, USA). The supernatant was then filtered through a 0.22 micrometer filter (Millex-GV, Merck Millipore, Burlington, MA, USA, Cat. No. SLGV033RS) and stored at −80 °C until further use, as detailed in the study by Venturini et al. (2020) [12].

### 4.4. Purification of Extracellular Vesicles (EVs)

For the isolation of extracellular vesicles (EVs), following the methodology described by Campos et al. (2023) with some modifications [49]. First, 100 mL of conditioned media derived from cultures of non-senescent or senescent HMEC-1 cells was collected. The samples were centrifuged at 2000× *g* for 30 min at 4 °C and filtered through 0.22 μm filters (Millex-GV, Merck Millipore, Cat. No. SLGV033RS). Subsequently, conditioned media were concentrated with Amicon Ultra-15 filters, 100 kDa MWCO (Merck Millipore, Cat. No. UFC910024), and centrifuged at 3000× *g* for 15 min at 4 °C. This protocol yielded 7–10 mL of concentrated conditioned media for each experimental condition. Finally, EVs were isolated using ExospinTM columns (Cell Guidance Systems, London, UK, Cat. No. EX01-50), following the manufacturer’s instructions. The EVs were stored at −80 °C until used, within a maximum storage time of one month.

### 4.5. Nanoparticle Tracking Analysis (NTA)

For the assessment of particle concentration and size distribution, nanoparticle tracking analysis (NTA) was carried out with a NanoSight NS300 (Malvern Panalytical Ltd., Malvern, UK), which evaluates Brownian motion of particles using a light scattering system. Samples of EVs obtained from non-senescent and senescent HMEC-1 cells were diluted 1:50 in 1 × PBS (final volume of 1 mL) to obtain 10–100 particles per image. The camera level was set to capture three videos per sample, with 30 s per video. Then, the videos were analyzed using the software Nanosight NTA 3.2 to determine the mean and mode of particle sizes (size distribution) and the number of particles per milliliter.

### 4.6. Scanning Electron Microscopy (SEM)

EVs derived from non-senescent and senescent HMEC-1 cells were fixed in 4% Paraformaldehyde/2% glutaraldehyde in 0.1 M PBS. Subsequently, EVs were incubated in 1% Osmium Tetroxide for 1 h and contrasted with 1% Uranium Acetate for another 1 h. An aliquot (10–20 µL) was spread on indium tin oxide-coated glass slides (SPI Supplies, West Chester, PA, USA). After drying, EVs were imaged (5670 × 3240 pixels) using a scanning electron microscope (Sigma—FESEM, Zeiss. Carl-Zeiss-Strasse 22, 73447 Oberkochen, Germany) equipped with a field emission cathode at 2 KeV accelerating voltage and 200 pA beam current at a magnification of 35K (1.9 nm pixel size). The sizes of the EVs were measured using Software ImageJ (Fiji, version 2.0.0-rc-67/1.52i, NIH), where the particles were identified and their dimensions calculated.

### 4.7. Determining Protein Concentration

Protein concentrations of total cell lysates and purified EVs were determined using the BCA Protein Assay Kit (Thermo Fisher Scientific, Waltham, MA, USA, Cat. No. 23225), following the manufacturer’s instructions. Briefly, each sample was diluted in RIPA buffer (Thermo Fisher Scientific, Waltham, MA, USA, Cat. No. 89901), containing protease and phosphatase inhibitors (Merck, Darmstadt, Germany, Cat. No. P8340 and Cat. No. P5726) in a range of 1:2 to 1:40, depending on sample type, and prepared in triplicate per assay. The standards (BSA) and the samples were added to a 96-well microplate, in triplicate, followed by the reagents in the kit (according to the manufacturer’s instructions). The absorbance measured at 562 nm wavelength was used to calculate the total protein concentration. The final concentration was determined after interpolating the curve values based on standard concentrations of BSA included in the assay.

### 4.8. Western Blotting

HMEC-1 cell lysates and purified EVs were further subjected to sonication in RIPA buffer containing protease and phosphatase inhibitors. Subsequently, 5–50 μg of proteins were heated/denatured in SDS-PAGE loading buffer, subjected to electrophoresis on 10% polyacrylamide gels, and then transferred to 0.45 μm nitrocellulose membranes (Santa Cruz Biotechnology, Santa Cruz, CA, USA, Cat. No. sc-3724). Next, membranes were blocked in 5% skimmed milk/0.1% Tween in 1 × PBS for 1 h, under constant shaking, and at room temperature. After blocking, the membranes were incubated overnight at 4 °C with primary antibodies diluted in blocking solution of 1% Tween-20 (*v*/*v*)/5% non-fat milk (*w*/*v*)/1 × TBS as indicated: anti-KDR (55B11) Rabbit mAb (Cell Signaling Technology, Danvers, MA, USA, Cat. No. 2479; 1:1000), anti-peNOS (BD Biosciences, San Jose, CA, USA, Cat. No. 612392), 1:1000), anti-eNOS (BD Biosciences, San Jose, CA, USA, Cat. No. 610299, 1:1000), anti-Nitrotyrosine (Merck Millipore, Burlington, MA, USA, Cat. No. 05-233, 1:1000), anti-FLT1 (C-17) (Santa Cruz Biotechnology, Santa Cruz, CA, USA, Cat. No. sc-316, 1:1000), anti-VEGF (C-1) (Santa Cruz Biotechnology, Santa Cruz, CA, USA, Cat. No. sc-7269, 1:1000), anti-CD81(B-11) (Santa Cruz Biotechnology, CA, USA, Cat. No. sc-166029. 1:1000), anti-Alix (1A12) (Santa Cruz Biotechnology, Santa Cruz, CA, USA, Cat. No. sc-53540; 1:1000), anti-TSG101 [EPR7130(B)] (Abcam, Cambridge, UK, Cat. No. ab125011 1:1000), anti-Calnexin (Novus Biologicals, Centennial, CO, USA, Cat. No. NB100-1965 1:3000), anti-β-Actin (Sigma-Aldrich, St. Louis, MO, USA, Cat. No. A5316, 1:5000). Subsequently, membranes were washed and incubated for 1 h at room temperature with secondary antibodies diluted in blocking solution as follows: Anti-Mouse IgG H&L (HRP) (Abcam, Cambridge, UK, Cat. No. ab205719, 1:5000) or anti-Rabbit (Anti-Rabbit IgG H&L (HRP) (Abcam, Cambridge, UK, Cat. No. ab205718; 1:5000). Afterwards, bound antibodies were detected with SuperSignalTM West Femto (Thermo Fisher Scientific, Waltham, MA, USA, Cat. No. 34094), according to the manufacturer’s instructions. Finally, the signals were visualized in an Omega LumTM G imaging system version V2.1.1027.0 (Gel Company, San Francisco, CA, USA).

### 4.9. Preparation of Washed Human Platelets

To prepare washed human platelets, we followed the protocol outlined by Mendez et al. (2020) [50]. Briefly, 20 mL of whole blood was collected and mixed at a 4:1 *v*/*v* ratio with Citrate–dextrose solution (ACD) (Sigma-Aldrich, St. Louis, MO, USA, Cat. No. C3821) as an anticoagulant. Platelet-rich plasma (PRP) was centrifuged at room temperature at 200× *g* for 10 min. After this step, PRP was transferred to a fresh tube and centrifuged at 900× *g* for 8 min at room temperature to obtain the platelet pellet. Next, the pellet was then resuspended in Calcium-Free Tyrode’s buffer (bioWORLD, Dublin, OH, USA, Cat. No. 40120249) diluted in ACD (at a 5:1 *v*/*v* ratio) and centrifuged at 900× *g* for another 8 min. The platelets were then resuspended in Calcium-Free Tyrode’s buffer, and their concentrations were determined using an automated hematology analyzer (BC-2800 Mindray Analyzer, Shenzhen, China). Concentrations of platelets were adjusted to 200–250 × 10^6^ platelets/mL for subsequent assays.

### 4.10. Cell Viability Assessed by Calcein-AM

To determine cell viability, the pre-adjusted washed platelets were incubated with 0.1 μM calcein-AM (UltraPure Grade, Thermo Fisher Scientific, Cat. No. 65-0853-39) for 20 min at 37 °C in the dark. Subsequently, EVs derived from senescent and non-senescent HMEC-1 cells, corresponding to 13 ng/µL, or the vehicle (1xPBS), were combined with calcein-labeled platelets in a 100 μL final reaction volume, and the mixture was further incubated for 10 min at 37 °C in the dark. Then, platelet populations and their viability were determined by FACS analysis (BD FACS Lyric Flow cytometer, BD, San Diego, CA, USA) using an anti-CD61 antibody, where the fraction (%) of calcein-negative platelets in the CD61+ subpopulation was defined as non-viable platelets, according to the method outlined previously [50]. Finally, platelets in 1 × PBS or 0.01% Triton X-100 were used as viable or dead controls, respectively.

### 4.11. Cytotoxic Activity Assessed by Release of LDH

For the cytotoxicity assay, the pre-adjusted washed platelets were exposed to vehicle (1 × PBS) or to EVs derived from non-senescent or senescent HMEC-1 cells (equivalent to 13 ng/µL) for 10 min at 37 °C. After this, platelets were centrifuged at 900× *g* for 8 min to obtain the supernatant and the working reagent of the lactate dehydrogenase (LDH) cytotoxicity kit (Cayman Chemical, Ann Arbor, MI, USA). Following that, the production of formazan (colored compound) was measured at 490 nm in a Thermo Scientific Multiskan GO, USA, as described previously [36]. Finally, platelets pre-incubated in 10% Triton X-100 were used as a control for maximum cytotoxicity.

### 4.12. Markers of Platelet Activation Assessed by Flow Cytometry

Washed platelets were treated with vehicle (1 × PBS) or EVs derived from non-senescent or senescent HMEC-1 cells (at a concentration of 13 ng/µL) for 10 min at 37 °C. Next, 30 μL aliquots of each condition were taken and incubated separately with antibodies directed against the surface markers P-selectin and activated GPIIb/IIIa (PAC-1). This step was followed by FACS analysis (BD FACS Lyric flow cytometer) and the mean fluorescence intensity (MFI) determination of subpopulations. Ultimately, the platelet population was identified with anti-CD61, as previously described [51].

### 4.13. Statistical Analyses

Data analysis was performed using IBM SPSS Statistics 26 and GraphPad Prism 9. All results were expressed as mean ± SEM. Before each analysis, normality and homoscedasticity were assessed in IBM SPSS Statistics 26 using the Shapiro–Wilk and Levene tests, respectively. Comparisons between groups were performed in GraphPad Prism 9 using Mann–Whitney, Welch’s *t*-test, Brown–Forsythe, and Welch ANOVA or two-way ANOVA, as indicated in the figure legends. For one-way and two-way ANOVA, pairwise comparisons were performed using Dunnett’s T3 and Bonferroni’s tests, respectively.

## Data Availability

The original contributions presented in this study are included in the article/Supplementary Materials. Further inquiries can be directed to the corresponding authors.

## Supplementary Materials

The following supporting information can be downloaded at https://www.mdpi.com/article/10.3390/ijms27020869/s1.
