# Supplementary material for: Extracellular Vesicles (EVs) Derived from Senescent Endothelial Cells Promote Platelet Activation
Source: Int J Mol Sci. 2026 Jan 15;27(2):869. doi: 10.3390/ijms27020869 (PMC12842113; doi:10.3390/ijms27020869)
Supplement: Supplementary file 1 [file ijms-27-00869-s001.zip › ijms-3942780-supplementary.pdf]

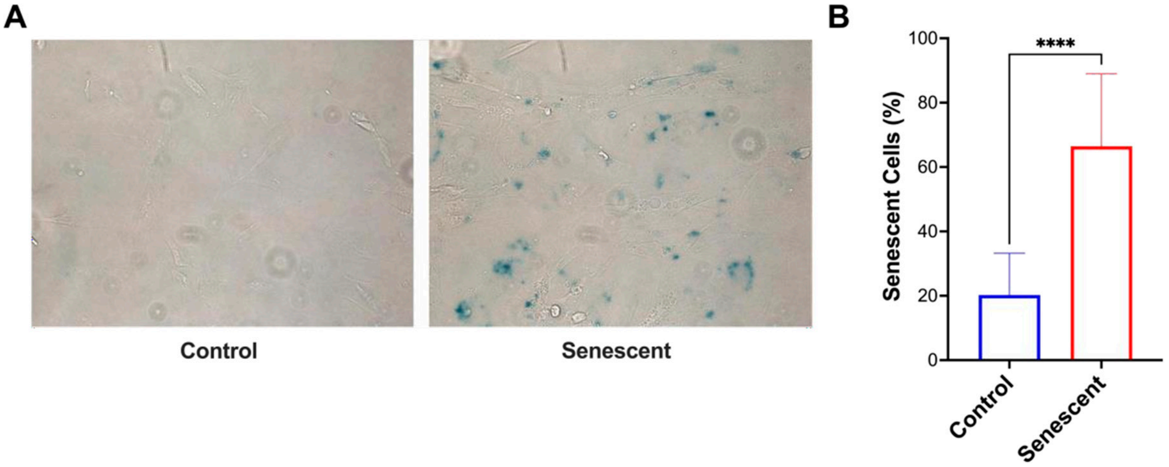

Figure S1. Doxorubicin-induced senescence in HMEC-1 cells. A) Representative images of SA-β-Gal staining in HMEC-1 cells after treatment with 0.05 μM doxorubicin for 72 hours. B) Quantification of SA-β-Gal activity in HMEC-1 cells treated with 0.05 μM doxorubicin for 72 hours. Error bars indicate mean ± SD of n = 3 (NS = no significant; * p < 0.05; ** p < 0.01; *** p < 0.001; t-student test).

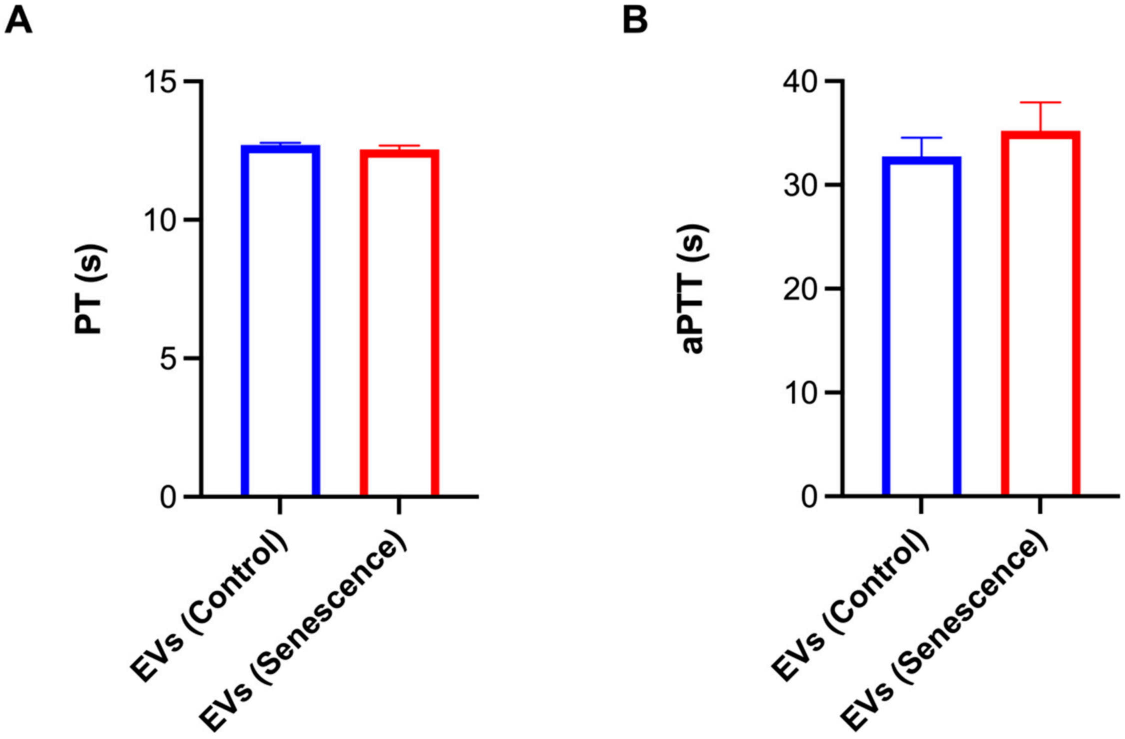

Figure S2. Coagulation Times After Exposure to Extracellular Vesicles from Senescent and Non-Senescent HMEC-1 Cells. Activated Partial Thromboplastin Time (aPTT) and Prothrombin Time (PT) were evaluated after incubating platelets with 13 ng/μL of extracellular vesicles (EVs) from senescent and non-senescent HMEC-1 cells. (A) PT and (B) aPTT results are shown, with non-senescent EVs represented by blue bars and senescent EVs by red bars.
